# Overexpression of HHLA2 in human clear cell renal cell carcinoma is significantly associated with poor survival of the patients

**DOI:** 10.1186/s12935-019-0813-2

**Published:** 2019-04-16

**Authors:** Lujun Chen, Dawei Zhu, Jun Feng, You Zhou, Qi Wang, Huijing Feng, Junping Zhang, Jingting Jiang

**Affiliations:** 1 Department of Tumor Biological Treatment, The Third Affiliated Hospital of Soochow University, Changzhou, 213003 Jiangsu China; 2 Jiangsu Engineering Research Center for Tumor Immunotherapy, The Third Affiliated Hospital of Soochow University, Changzhou, 213003 Jiangsu China; 3 Department of Oncology, Shanxi Academy of Medical Sciences, Shanxi Dayi Hospital, Taiyuan, 030032 Shanxi China; 4 Institute of Cell Therapy, Soochow University, Changzhou, 213003 Jiangsu China

**Keywords:** HHLA2, Clear cell renal cell carcinoma, Prognosis, Epithelial-to-mesenchymal transition

## Abstract

**Background:**

It is well known that human clear cell renal cell carcinoma (ccRCC) is a highly immunogenic and chemo-resistant tumor. Recently, emerging data suggest that the immune checkpoint blockade therapy is an important breakthrough in the treatment against ccRCC. HHLA2, a recently reported member of B7 family, is uniquely expressed in humans but not in mice, and it plays an important role in the functional inhibition of CD4 and CD8 T cells. Herein, we aimed to study the clinical implications of HHLA2 expression in human ccRCC and its potential regulatory role in the biological functions of the cancer cells.

**Methods:**

In the present study, we examined HHLA2 expression in human ccRCC tissues and analyzed the clinical implications as well as prognostic value. The intervention of HHLA2 in human ccRCC cell lines ACHN and 786-O was performed and its effect on the cellular function of the cells was also analyzed. We also identified the differentially expressed genes upon HHLA2 knockdown in ccRCC cell lines by using gene microarray analysis.

**Results:**

We found that higher HHLA2 mRNA expression level in human ccRCC tissues compared with that in adjacent normal tissues based on TCGA data, and the HHLA2 expression at mRNA level was positively and significantly correlated with PD-L1, PD-L2, B7-H6, but negatively and significantly correlated with B7-H3. Moreover, our immunohistochemistry study showed that the staining intensity of HHLA2 in human ccRCC tissues was significantly higher than that in the adjacent normal tissues, and the overall survival rate of ccRCC patients with higher HHLA2 expression was significantly poorer than that of the patients with lower HHLA2 expression. Higher expression of HHLA2 in ccRCC tissues was positively and significantly associated with larger tumor size and advanced TNM stage. The COX model revealed that the parameters including patient’s age, TNM stage and HHLA2 expression level could be used as the independent risk factors respectively for the prognostic prediction of the patients. Our cellular study showed that upon knockdown of HHLA2 expression in human ccRCC cell lines, the cell viability, the migration and the invasion ability were significantly inhibited, while the cell cycle arrest at G1 phase was induced and the expressions of Cyclin D1, c-Myc and Cyclin E1 were decreased. In addition, according to the microarray data, the expressions of epithelia-to-mesenchymal transition markers, such as E-cadherin, N-cadherin and Vimentin, were significantly changed after knockdown of HHLA2 expression.

**Conclusions:**

Our findings indicated that HHLA2 was involved in the progression of human ccRCC and could be used as an important prognostic predictor for this malignancy.

## Background

Renal cell carcinoma (RCC) has been found to be the most common type of kidney cancers in adults [1]. Although there are many uncommon histological subtypes of renal cell carcinoma, the common histological subtypes of RCC can be majorly classified as clear cell renal cell carcinoma (ccRCC), papillary renal cell carcinoma (pRCC) and chromophobe renal cell carcinoma (chRCC) [2, 3]. Among these subtypes, the ccRCC is derived from the proximal convoluted tubule, and accounts for 80–90% of all RCC cases. The surgical managements, including nephrectomy and partial nephrectomy, still remain as the most recommended treatments for RCC [4]. Moreover, percutaneous ablation therapy, chemotherapy, targeted therapy, and even immunotherapy, have been recently used to treat such malignancy [5–8]. However, the 5-year survival rate of ccRCC patients still remains to be improved, therefore, it’s urgently necessary to develop novel diagnostic and therapeutic targets against ccRCC.

It is well known that RCC is a highly immunogenic and chemo-resistant tumor [9, 10]. Although the anti-angiogenic therapies have significantly improved the overall survival of the patients with advanced or metastatic ccRCC over the past decade, the immune checkpoint blockade therapy (ICBT), such as anti-programmed cell death 1 (anti-PD-1) therapy, has also been shown to be efficacious in metastatic RCC patients once anti-angiogenic therapy fails [11–13]. Emerging data on the mechanisms of reversing immuno-suppression in tumor microenvironment have led to the development of novel ICBT strategies in RCC patients, such as combination therapy of Nivolumab (anti-PD-1) plus Ipilimumab (anti-CTLA-4), or even ICBT in combination with targeted therapies [9, 14, 15]. All these findings indicate that the blockade of negative regulatory co-stimulatory molecules mediating signal pathways has been widely accepted as an important breakthrough in the treatment of RCC [16]. Moreover, some negative regulatory co-stimulatory molecules such as B7 family ligands B7-H3 and B7-H4, have been found abnormally expressed in human RCC tissues, and their expression levels in cancer tissues are significantly associated with cancer progression and poor prognosis, indicating that such negative regulatory co-stimulatory molecules can be used as important biomarkers and therapeutic targets against RCC [17, 18].

Recently, certain novel B7 family ligands have been characterized and reported to be involved in tumor immune evasion [19–21]. Human endogenous retrovirus-H long terminal repeat-associating protein 2 (HHLA2, also known as B7-H7), a recently reported member of B7 family, is uniquely expressed in humans but not in mice, and plays an important role in the functional inhibition of CD4 and CD8 T cells [22]. The TMIGD2 (transmembrane and immunoglobulin domain containing 2), a single-pass type I membrane protein containing one immunoglobulin-like domain, has been characterized as the specific receptor for HHLA2 [23]. Several studies have shown that higher expression of HHLA2 in human cancer tissues is significantly associated with cancer progression and poor prognoses of the patients [24, 25]. In the present study, we aimed to examine the expression pattern of HHLA2 in human ccRCC tissues and to analyze its clinical significance. And we further investigated the contribution of HHLA2 to the biological function of ccRCC cell lines and the progression of this malignancy.

## Materials and methods

### Patients and tissue samples

The ccRCC tissue-array (Catalog: HKidE180Su03, Shanghai Outdo Biotech Co., Ltd., Shanghai, P. R. China) was used in the present study. A total of 90 patients (59 males and 31 females, aged 29 to 82 years) who underwent surgery between October 2006 and February 2008 were enrolled in this study. Incomplete tissue samples and several missing tissue samples were excluded during the heat-induced antigen retrieval, and finally a total of 87 cases were involved in the present work. The detailed clinical parameters of the patients were shown in Table 1. All patients gave informed consent for participation, and the protocol for the present study was approved by the ethics committee of the Third Affiliated Hospital of Soochow University.

**Table 1:** Association between the HHLA2 expression in ccRCC tissues and the patients’ clinical parameters

Clinical parameters	Cases	HHLA2 expression level		*χ* ^*2*^	*P*
		Low (H-score ≤ 90)	High (H-score > 90)		
Gender^a^				0.278	0.598
Male	56	38	18		
Female	30	22	8		
Age (years)^a^				0.059	0.809
≤ 60	48	34	14		
> 60	38	26	12		
Tumor size (cm)				4.155	*0.042*
≤ 7	66	50	16		
> 7	21	11	10		
TNM stage				4.161	*0.041*
I + II	81	59	22		
III + IV	6	2	4		

Italics signifies *P *< 0.05

^a^One case missing data was excluded

### Antibodies and major reagents

Rabbit anti-human HHLA2 polyclonal antibody (LS-C321945, Lifespan Biosciences), monocloncal mouse anti-human CD8 (Clone C8/144B, Dako), monocloncal mouse anti-human CD34 (ZM-0046, Zhongshan GoldenBridge Biotechnology), HRP-conjugated goat anti-mouse/rabbit secondary antibody (K500711, Dako), rabbit anti-human GAPDH antibody (Sigma), SYBR Green Master Mix kits (TaKaRa), DMEM and fetal bovine serum (Gibco) were used in the present study.

### Immunohistochemical staining and the assessment of staining intensity

Immunohistochemical staining was performed as described in our previous studies [26, 27]. In brief, the antigen retrieval was done by heating the tissue sections at 100 °C in EDTA solution (pH 9.0) for 30 min. The sections were incubated with primary antibody against HHLA2 at 4 °C overnight, followed by incubation with HRP-conjugated secondary antibody. Diaminobenzene was used as the chromogen, and hematoxylin was used as the nuclear counterstain. Finally the sections were dehydrated, cleared and mounted. Moreover, the assessment of the immunostaining intensity of HHLA2 was done by using the *H*-*score* method in our published reports [26, 28–31].

### RNA interference (RNAi), cell culture and treatments

The stable cell lines were established by using RNAi approach. Small hairpin RNA (shRNA) against human HHLA2 gene (NM_007072.2; GenBank) was obtained from Shanghai Generay Biotech Co., Ltd. (Shanghai, China). The shRNA target sequences against HHLA2 were as follows, shRNA-1: 5′-GCCAAGAAACAGCTTCCCATA-3′; and shRNA-2: 5′-CCTGGATGTTAAGGATTCCAA-3′. The non-targeted control sequence was used as previously described [28–30]. The shRNA was cloned into a lentiviral vector encoding green fluorescent protein (GFP) gene. The human ccRCC cell lines 786-O and ACHN (Chinese Academy of Sciences, Shanghai Institutes for Biological Sciences) were cultured in standard DMEM supplemented with 10% fetal bovine serum under standard culture conditions (5% CO_2_, 37 °C). Recombinant HHLA2-targeting lentivirus (LV-HHLA2-shRNA virus) or control mock lentivirus (LV-NC virus) were transfected into 786-O and ACHN cells. Then the GFP-positive cells were subsequently sorted from the transfected cells in a flow sorter (Aria II, BD, USA).

### RNA isolation and real-time PCR (RT-PCR)

The knockdown of HHLA2 expression at mRNA level in the two ccRCC cell lines ACHN and 786-O was confirmed using RT-PCR. The primer sequences of human HHLA2 were as follows: forward, 5′-GGAACACTTCATTTTCCCCAATTC-3′ and reverse, 5′-TCTCCTACATGCTCTCCTTCCT-3′. The sequences of the primers for reference gene human *GAPDH*, and the detailed protocol for real-time PCR were described in detail in our published reports [28–30].

### Cellular studies of proliferation, migration, invasion and cell cycle analysis under HHLA2 knockdown

The cell proliferation ability was examined using Cell Counting Kit-8, the cell migration ability was assessed using a wound scrape assay, and the cell invasive ability was examined by using matrigel-coated invasion chambers [29, 30]. For the cell cycle assay, the cells from different groups were inoculated in 6-well plates and cultured for 48 h, then were washed with ice-cold PBS and fixed in 70% (v/v) ice-cold ethanol solution overnight at 4 °C, then in the following day, these cells were analyzed by using flow cytometry according to the instruction of cell cycle analysis kit (Sigma, MO, USA), the cell cycle information was analyzed using ModFit LT 4.0 software.

### Western blotting analysis

The Western blotting analysis was performed according to the protocol we have reported [28–30]. The anti-HHLA2 (1:2000; Abcam, MA, USA), the anti-GAPDH (1:4000, Sigma, St. Louis, MO, USA), and the HRP-labeled goat anti-mouse/rabbit secondary antibody (1:6000, Sigma Aldrich, St. Louis, MO, USA) were used, and the immunoreaction was visualized using an enhanced chemiluminescence detection kit (Thermo Fisher, MA, USA) and exposed to X-ray film, and band densities were quantified by densitometry with a video documentation system (Gel Doc 2000, Bio-Rad).

### Agilent lncRNA microarray analysis

The Agilent Human lncRNA Microarrays (V6) were used on the Agilent Microarray-Based Gene Expression Analysis platform by Oebiotech Co. (Shanghai, P.R. China) as described previously [30, 32]. In brief, the total RNA was extracted from 786-O and ACHN cells from LV-HHLA2-shRNA (sh1) and LV-NC groups, and then quantified by the NanoDrop ND-2000 (Thermo Scientific) and the RNA integrity was assessed by Agilent Bioanalyzer 2100 (Agilent Technologies). The total RNA were reverse-transcribed to double strand cDNA, then synthesized into cRNA and labeled with Cyanine-3-CTP. The labeled cRNAs were hybridized onto the microarray. After washing, the arrays were scanned by the Agilent Scanner G2505C (Agilent Technologies). The Feature Extraction software (Version10.7.1.1, Agilent Technologies) was used to analyze array images to get raw data. Genespring (Version 13.1, Agilent Technologies) was employed to finish the basic analysis with the raw data. To begin with, the raw data was normalized with the quantile algorithm. The probes that at least 1 condition out of 2 conditions has flags in “P” were chosen for further data analysis. Differentially expressed genes or lncRNAs were then identified through fold change. The threshold set for up- and down-regulated genes was a fold change > = 2.0. Afterwards, GO analysis and KEGG analysis were applied to determine the roles of these differentially expressed mRNAs.

### Statistical analysis

GraphPad Prism 5.0 software package (GraphPad Software, Inc., San Diego, USA) was used in the present statistical analysis, and the paired Student’s *t* test, the Wilcoxon signed-rank test, the Chi square test or the Log-rank test was used where appropriate. A *P* value < 0.05 was considered as statistically significant.

## Results

### Survey of HHLA2 expression at the mRNA level in human ccRCC tissues based on TCGA data

**Fig. 1:**
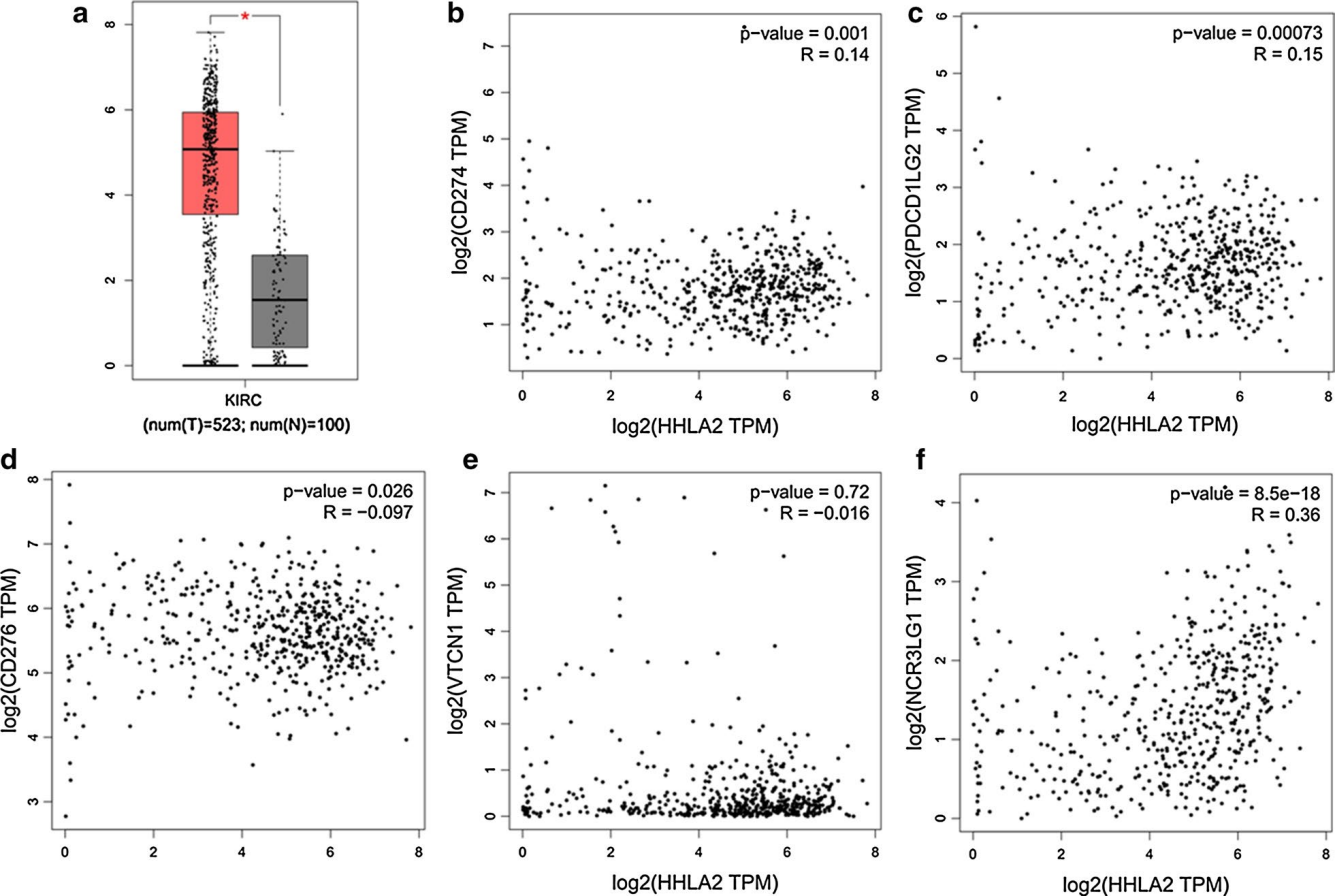
Survey of HHLA2 expression at mRNA level in human ccRCC tissues based on TCGA data. We compared the HHLA2 expression at mRNA level between human ccRCC tissues and adjacent normal tissues according to TCGA data from http://gepia.cancer-pku.cn/. **a** Higher expression in human ccRCC tissues was found compared with adjacent normal tissues (*P *< 0.05). The HHLA2 mRNA expression level was positively and significantly correlated with PD-L1 (**b**, *P *< 0.05), PD-L2 (**c**, *P *< 0.001), B7-H6 (**f**, *P *< 0.0001), but negatively and significantly correlated with B7-H3 (**d**, *P *< 0.05), and there was no significant association between HHLA2 and B7-H4 (**e**, *P *> 0.05)

According to TCGA data from http://gepia.cancer-pku.cn/, we firstly compared the HHLA2 expression at the mRNA expression level between human ccRCC tissues and adjacent normal tissues, and higher expression of HHLA2 was found in human ccRCC tissues compared with the adjacent normal tissues (Fig. 1a, *P *< 0.05). Secondly, because HHLA2 is an important member of B7 family ligands, we then assessed the associations between the HHLA2 expression at mRNA level and other members from B7 family in human ccRCC tissues. We found that the mRNA expression level of HHLA2 was positively and significantly correlated with PD-L1 (Fig. 1b, *P *< 0.05), PD-L2 (Fig. 1c, *P *< 0.001) and B7-H6 (Fig. 1f, *P *< 0.0001), while it was negatively and significantly correlated to B7-H3 (Fig. 1d, *P *< 0.05), and there was no association between HHLA2 and B7-H4 (Fig. 1e, *P *> 0.05), suggesting a certain similarity of the potential biological function of HHLA2 with other immune checkpoint molecules in the tumor microenvironment of human ccRCC.

### Immunostaining of HHLA2 expression in human ccRCC tissues and its clinical implications

**Fig. 2:**
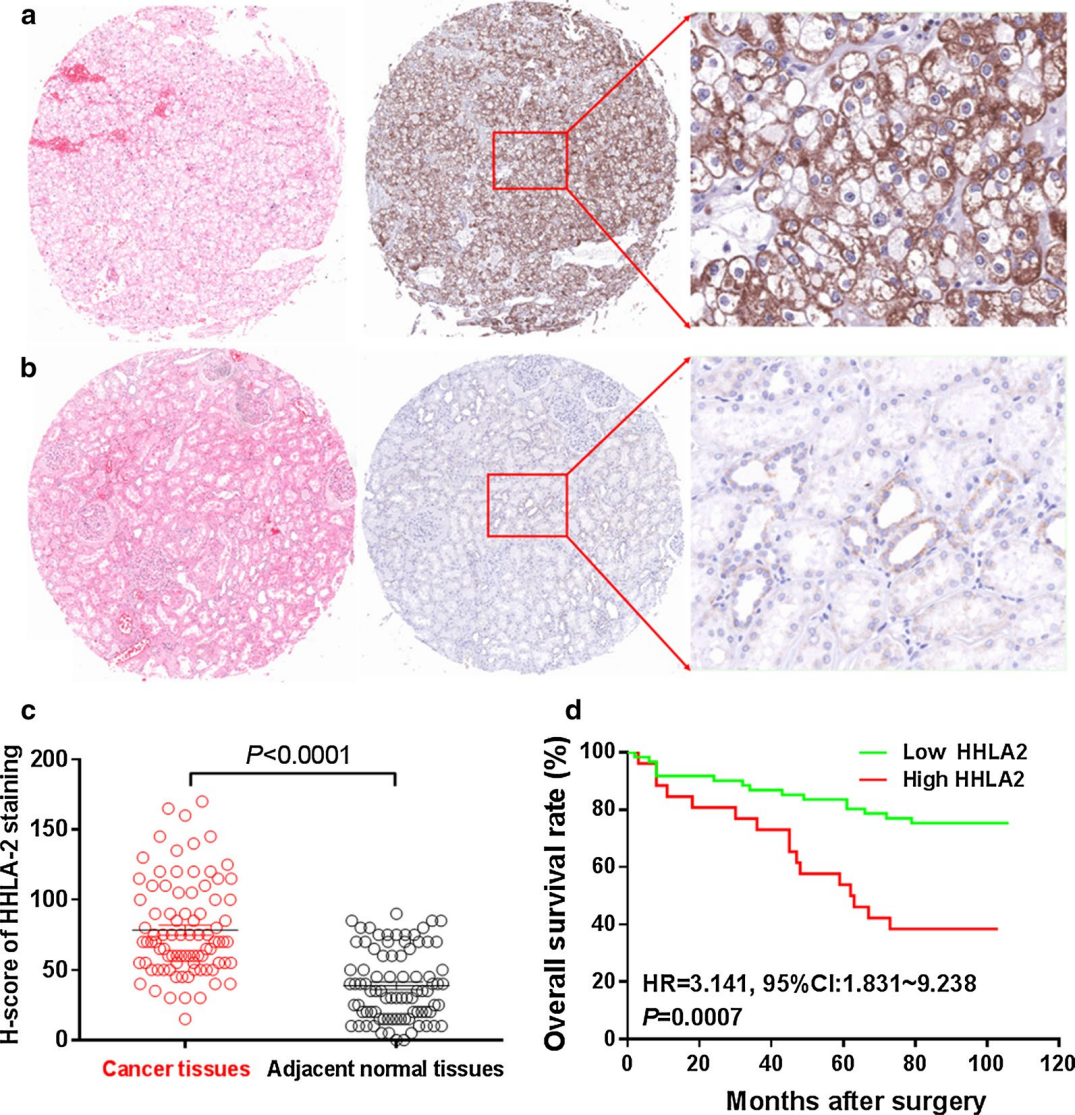
HHLA2 expression in human ccRCC tissues. **a** The positive staining of HHLA2 could be predominantly found in the cytoplasm and on the membrane of the cancer cells in ccRCC tissues. **b** Weak staining of HHLA2 was found in adjacent normal tissues. **c** The staining intensity of HHLA2 in human ccRCC tissues was significantly higher than that in adjacent normal tissues (*P *< 0.0001). **d** The overall survival rate of ccRCC patients with low HHLA2 expression was significantly higher than that of the patients with high HHLA2 expression (HR = 3.141, 95% CI 1.831–9.238, *P *= 0.007)

In order to study the expression pattern of HHLA2 in human ccRCC tissues and its clinical implications, we also performed the immunohistochemistry assay to examine the HHLA2 expression in human ccRCC tissue array. As shown in Fig. 2a, the positive staining of HHLA2 was predominantly found in the cytoplasm and on the membrane of the cancer cells. Similar with the expression pattern of HHLA2 at mRNA expression level from TCGA data, the staining intensity of HHLA2 in human ccRCC tissues was significantly higher than that in adjacent normal tissues (Fig. 2c), and the overall survival rate of ccRCC patients with low HHLA2 expression was significantly higher than that of the patients with high HHLA2 expression (HR = 3.141, 95% CI 1.831–9.238, *P *= 0.007, Fig. 2d). As shown in Table 1, we also found that the higher expression of HHLA2 was positively and significantly associated with lager tumor size (*P *= 0.042), and advanced TNM stage (*P *= 0.041), but we could not find any association with patient’s gender and age. Moreover, as shown in Table 2, the COX model also showed that the parameters including patient’s age, TNM stage and the HHLA2 expression level could be used as the independent risk factors for the prognostic prediction of the patients. Interestingly, we also perform the CD8 and the CD34 staining, which reflected the tumor infiltrating CD8^+^ T cells and the intratumoral microvessels respectively in ccRCC tissues, and our statistical data showed that there weren’t any significant associations between HHLA2 expression in ccRCC tissues and the infiltrating intensity of CD8^+^ T cells, or between HHLA2 expression in ccRCC tissues and the intratumoral microvessels (data not shown). Therefore, our present results unveil that the abnormal expression of HHLA2 might promote the cancer progression in certain biological process but not via the regulation of CD8^+^ T cell infiltration or intratumoral angiogenesis.

**Table 2:** Cox model analysis for the association between the HHLA2 expression level and patients’ clinical parameters

Variables	Univariate analysis		Multivariate analysis	
	HR (95% CI)	*P*	HR (95% CI)	*P*
Gender (male: female)	0.923 (0.448–1.901)	0.828	1.415 (0.637–3.145)	0.394
Age (> 60: ≤ 60 years)	2.971 (1.397–6.320)	*0.005*	3.504 (1.578–7.784)	*0.002*
Tumor size (> 7: ≤ 7 cm)	2.757 (1.347–5.643)	*0.005*	1.098 (0.444–2.715)	0.840
TNM stage (II + III + IV: I)	3.762 (1.836–7.705)	*0.000*	3.745 (1.514–9.260)	*0.007*
HHLA2 expression (high: low)	3.179 (1.565–6.456)	*0.001*	3.128 (1.510–6.487)	*0.002*

Italics signifies *P *< 0.05

### Establishment of stable knockdown of HHLA2 expression in ccRCC cell lines 786-O and ACHN

**Fig. 3:**
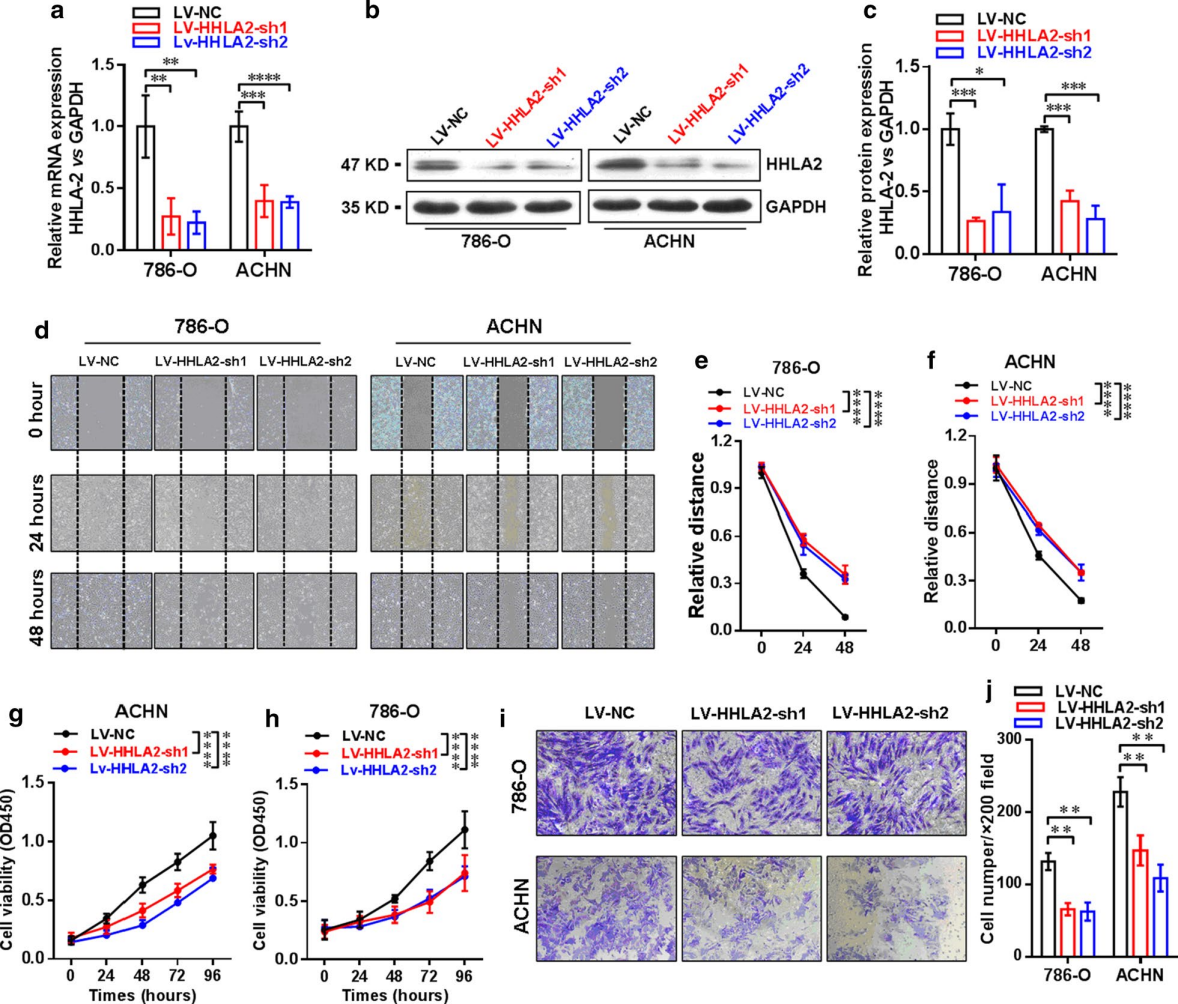
Establishment of stable knockdown of HHLA2 expression in ccRCC cell lines 786-O and ACHN. **a** The knockdown efficiency was confirmed by real-time RT-PCR analysis, and the data showed that the mRNA expression level of HHLA2 was significantly decreased in the LV-HHLA2-sh1 (*P *< 0.01 in 786-O cells, and *P *< 0.001 in ACHN cells) and LV-HHLA2-sh2 (*P *< 0.01 in 786-O cells, and *P *< 0.0001 in ACHN cells) compared with LV-NC group cells respectively. **b**, **c** By using the western blotting analysis, we also confirmed that the HHLA2 expression at the protein level was significantly decreased in the LV-HHLA2-sh1 (*P *< 0.001 in 786-O cells, and *P *< 0.001 in ACHN cells) and LV-HHLA2-sh2 (*P *< 0.05 in 786-O cells, and *P *< 0.001 in ACHN cells) compared with LV-NC group cells. **d**–**f** The migration ability of the ccRCC cell lines after knockdown HHLA2 expression in the LV-HHLA2-sh1 and LV-HHLA2-sh2 groups was examined compared with the LV-NC group cells. At the time point of 48 h, the relative distance of LV-HHLA2-sh1 (*P *< 0.0001 in 786-O cells, and *P *< 0.0001 in ACHN cells) and LV-HHLA2-sh2 (*P *< 0.0001 in 786-O cells, and *P *< 0.0001 in ACHN cells) was significantly increased compared with the LV-NC group cells. **g**, **h** Our CCK-8 assay showed that the cell proliferation ability of ccRCC cell lines was significantly decreased in the LV-HHLA2-sh1 (*P *< 0.0001 in 786-O cells, and *P *< 0.0001 in ACHN cells) and LV-HHLA2-sh2 (*P *< 0.0001 in 786-O cells, and *P *< 0.0001 in ACHN cells) compared with the LV-NC group cells. **i**, **j** Transwell assay showed that the cell invasive ability of ccRCC cell lines was significantly decreased in the LV-HHLA2-sh1 (*P *< 0.01 in 786-O cells, and *P *< 0.01 in ACHN cells) and LV-HHLA2-sh2 (*P *< 0.01 in 786-O cells, and *P *< 0.01 in ACHN cells) compared with the LV-NC group cells

In order to further investigate the biological function of HHLA2 in human ccRCC cell lines, we establish the stable knockdown expression of HHLA2 in ccRCC cell lines 786-O and ACHN by using shRNA via lentiviral infection and cell sorting by using detecting GFP expression in the flow sorter. Then, we check the knockdown efficiency by real-time RT-PCR analysis, and our data showed that the mRNA expression level of HHLA2 was significantly decreased in the LV-HHLA2-sh1 (*P *< 0.01 in 786-O cells, and *P *< 0.001 in ACHN cells) as well as the LV-HHLA2-sh2 (*P *< 0.01 in 786-O cells, and *P *< 0.0001 in ACHN cells) group compared with the LV-NC group (Fig. 3a). Consistently, we also found that the HHLA2 expression at the protein level was significantly decreased in the LV-HHLA2-sh1 (*P *< 0.001 in 786-O cells, and *P *< 0.001 in ACHN cells) and LV-HHLA2-sh2 (*P *< 0.05 in 786-O cells, and *P *< 0.001 in ACHN cells) groups compared with the LV-NC group (Fig. 3b, c).

Then, we further assessed the migration ability of the ccRCC cell lines after knockdown HHLA2 expression in the LV-HHLA2-sh1 as well as LV-HHLA2-sh2 group in contrast to the LV-NC group, and as shown in Fig. 3d–f, at the time point of 48 h, the relative distance of LV-HHLA2-sh1 (*P *< 0.0001 in 786-O cells, and *P *< 0.0001 in ACHN cells) as well as LV-HHLA2-sh2 (*P *< 0.0001 in 786-O cells, and *P *< 0.0001 in ACHN cells) group was significantly increased in contrast to the LV-NC group, suggesting that knockdown of HHLA2 expression significantly reduce the migration ability of ccRCC cell lines. Our CCK-8 assay also showed that the cell proliferation ability of ccRCC cell lines was significantly decreased in the LV-HHLA2-sh1 (*P *< 0.0001 in 786-O cells, and *P *< 0.0001 in ACHN cells) and LV-HHLA2-sh2 (*P *< 0.0001 in 786-O cells, and *P *< 0.0001 in ACHN cells) groups compared with the LV-NC group (Fig. 3g, h). Our transwell assay also showed that the cell invasive ability of ccRCC cell lines was significantly decreased in the LV-HHLA2-sh1 (*P *< 0.01 in 786-O cells, and *P *< 0.01 in ACHN cells) and LV-HHLA2-sh2 (*P *< 0.01 in 786-O cells, and *P *< 0.01 in ACHN cells) groups compared with the LV-NC group (Fig. 3i, j).

### Knockdown of HHLA2 significantly decreased the expressions of Cyclin D1, c-Myc and Cyclin E1 in ccRCC cell lines

**Fig. 4:**
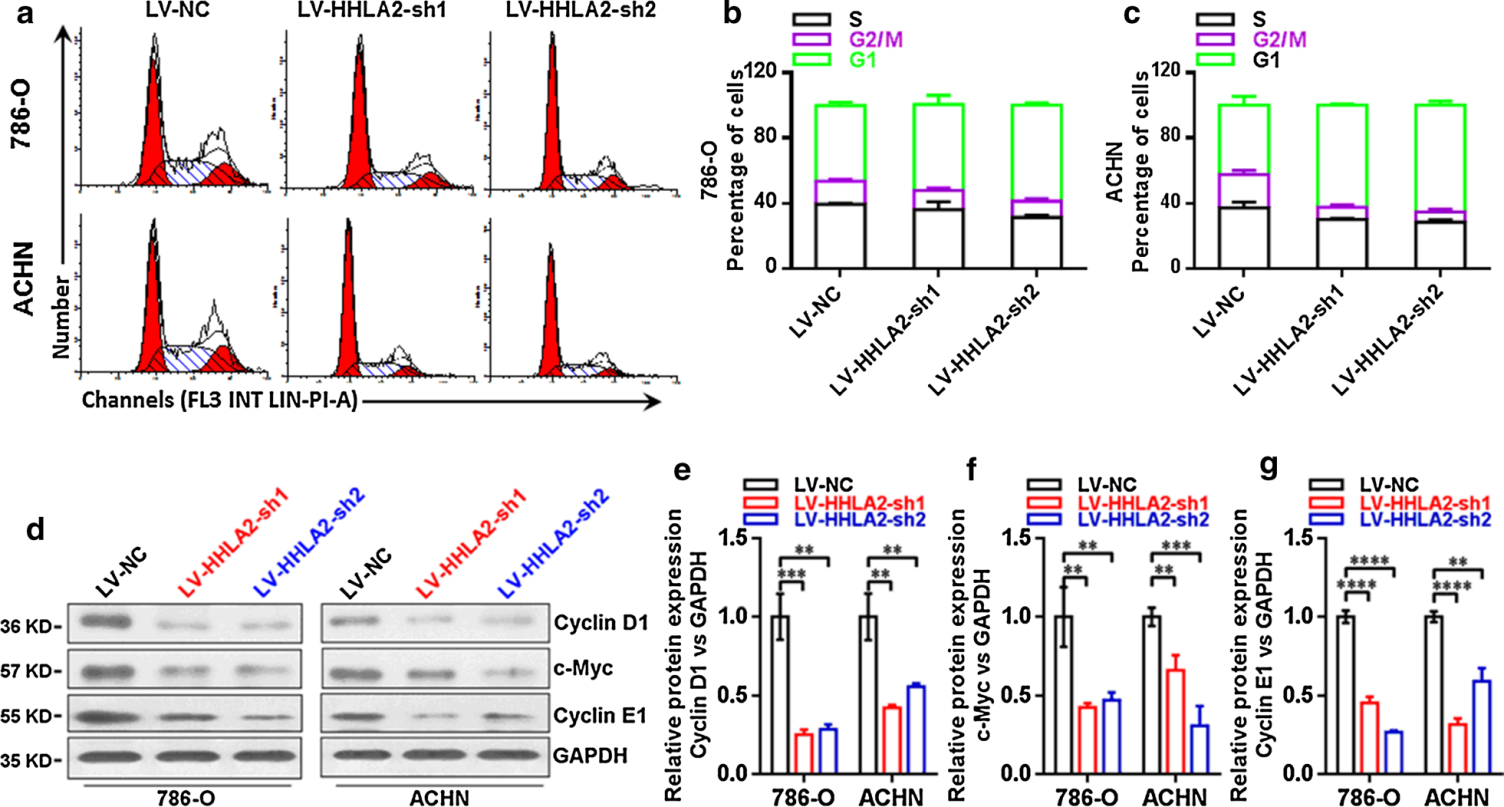
Expressions of Cyclin D1, c-Myc and Cyclin E1 in ccRCC cell lines after knockdown of HHLA2. **a**–**c** The knockdown HHLA2 expression significantly increased the ratio of G1 phase and induced cell cycle arrest in human ccRCC cell lines. **d** Western blotting analysis of Cyclin D1, c-Myc and Cyclin E1 in ccRCC cell lines after knockdown of HHLA2 expression in 786-O and ACHN cells respectively. **e** The Cyclin D1 was significantly decreased in the LV-HHLA2-sh1 (*P *< 0.01 in 786-O cells, and *P *< 0.01 in ACHN cells) and LV-HHLA2-sh2 (*P *< 0.01 in 786-O cells, and *P *< 0.01 in ACHN cells) compared with the LV-NC group cells. **f** The c-Myc was significantly decreased in the LV-HHLA2-sh1 (*P *< 0.01 in 786-O cells, and *P *< 0.01 in ACHN cells) and LV-HHLA2-sh2 (*P *< 0.01 in 786-O cells, and *P *< 0.001 in ACHN cells) compared with the LV-NC group cells. **g** The Cyclin E1 was significantly decreased in the LV-HHLA2-sh1 (*P *< 0.0001 in 786-O cells, and *P *< 0.0001 in ACHN cells) and LV-HHLA2-sh2 (*P *< 0.0001 in 786-O cells, and *P *< 0.01 in ACHN cells) compared with the LV-NC group cells

As shown in Fig. 4a–c, the knockdown expression of HHLA2 significantly increased the ratio of G1 phase and induced cell cycle arrest in human ccRCC cell lines, we then further examined the expressions of Cyclin D1, c-Myc and Cyclin E1 at the protein level in knockdown group cells in contrast to the control group cells. And as shown in Fig. 4d–g, our data showed that, the Cyclin D1 was significantly decreased in the LV-HHLA2-sh1 (*P *< 0.01 in 786-O cells, and *P *< 0.01 in ACHN cells) as well as LV-HHLA2-sh2 (*P *< 0.01 in 786-O cells, and *P *< 0.01 in ACHN cells) group in contrast to the LV-NC group, the c-Myc was significantly decreased in the LV-HHLA2-sh1 (*P *< 0.01 in 786-O cells, and *P *< 0.01 in ACHN cells) as well as LV-HHLA2-sh2 (*P *< 0.01 in 786-O cells, and *P *< 0.001 in ACHN cells) group in contrast to the LV-NC group, the Cyclin E1 was significantly decreased in the LV-HHLA2-sh1 (*P *< 0.0001 in 786-O cells, and *P *< 0.0001 in ACHN cells) as well as LV-HHLA2-sh2 (*P *< 0.0001 in 786-O cells, and *P *< 0.01 in ACHN cells) group in contrast to the LV-NC group.

### HHLA2 was potentially involved in the promotion of epithelial-to-mesenchymal transition (EMT) in human ccRCC cells

**Fig. 5:**
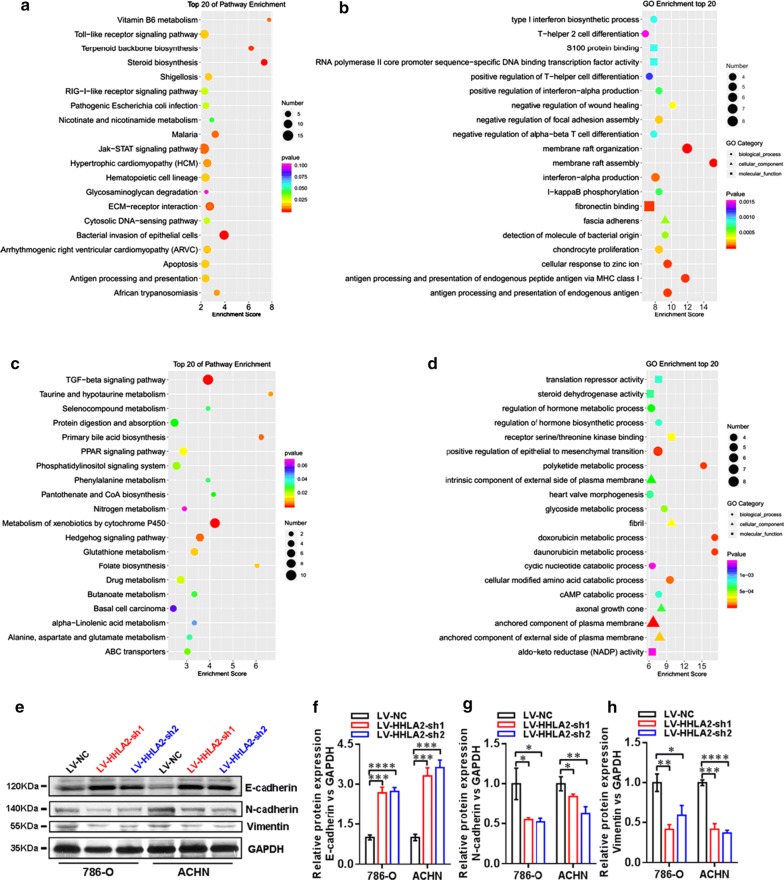
HHLA2 is potentially involved in the regulation of EMT in human ccRCC cells. We identified the differentially expressed gene profiles between LV-HHLA2-sh1 and LV-NC groups of ccRCC cell lines using microarray analysis. **a**, **b** The co-up-regulated genes were involved in the KEGG and GO analyses, and the top 20 pathways were listed, and for the co-down-regulated genes, the top 20 pathways were listed in **c**, **d**. In the following cellular study, we further examined the changes of EMT markers after HHLA2 knockdown expression in ccRCC cell lines. **e** Western blotting analysis of E-cadherin, N-cadherin and Vimentin in 786-O and ACHN cells in different groups. **f** After knockdown of HHLA2 in 786-O and ACHN, the expression of E-cadherin was significantly increased (In 786-O: LV-HHLA2-sh1 vs LV-NC: *P *< 0.001, LV-HHLA2-sh2 vs LV-NC: *P *< 0.0001; In ACHN: LV-HHLA2-sh1 vs LV-NC: *P *< 0.001, LV-HHLA2-sh2 vs LV-NC: *P *< 0.001). **g** After knockdown of HHLA2 in 786-O and ACHN, the expression of N-cadherin was significantly decreased (In 786-O: LV-HHLA2-sh1 vs LV-NC: *P *< 0.05, LV-HHLA2-sh2 vs LV-NC: *P *< 0.05; In ACHN: LV-HHLA2-sh1 vs LV-NC: *P *< 0.01, LV-HHLA2-sh2 vs LV-NC: *P *< 0.05). **h** After knockdown of HHLA2 in 786-O and ACHN, the expression of Vimentin was significantly decreased (In 786-O: LV-HHLA2-sh1 vs LV-NC: *P *< 0.01, LV-HHLA2-sh2 vs LV-NC: *P *< 0.05; In ACHN: LV-HHLA2-sh1 vs LV-NC: *P *< 0.001, LV-HHLA2-sh2 vs LV-NC: *P *< 0.0001)

In order to further investigate the potential contribution of HHLA2 to cellular function and signal pathways of human ccRCC cells in cancer progression, we performed the Agilent lncRNA microarray analysis to identify the differentially expressed genes profiles between LV-HHLA2-sh1 and LV-NC groups. As shown in Fig. 5a, b, the co-up-regulated genes were involved in the KEGG and GO analyses, and the top 20 pathways were listed, and for the co-down-regulated genes, the top 20 pathways were also listed in Fig. 5c, d. Therefore, the data from the present microarray analysis also support the conclusion from the cellular function study after knockdown of HHLA2 expression in certain pathways. For example, we found that the positive regulation of EMT was involved in the GO enrichment of top 20 pathways of co-down-regulated gene profiles (Fig. 5d). Then, we further performed the study to examine the change of EMT markers after HHLA2 knockdown expression in ccRCC cell lines, including E-cadherin, N-cadherin and Vimentin (as shown in Fig. 5e). The statistical results showed that, after knockdown of HHLA2 in 786-O and ACHN, the expression of E-cadherin was significantly increased (Fig. 5f), the expressions of N-cadherin (Fig. 5g) and Vimentin (Fig. 5h) were significantly decreased.

## Discussion

HHLA2 was first characterized by Mager and his colleagues in 1999, and was then named as HHLA2 due to its long terminal repeats in 3′-UTR [33]. Human *HHLA2* is located in the 3q13.13, which is very close to *B7*-*1* and *B7*-*2* genes, and shows high homology to *PD*-*L1* [22]. As an important co-stimulatory molecule in the negative regulation of T cells response, HHLA2 has been found to be widely expressed in antigen-presenting cells and T cells, but weakly expressed in resting dendritic cells and macrophages [22]. The transmembrane and immunoglobulin domain containing 2 (TMIGD2) is the receptor of HHLA2, which could be found in naive T cells and NK cells, as well as some endothelial cells and epithelial cells [34].

It has been demonstrated that over-expression of HHLA2 in tumor microenvironment could dampen the T-cell mediated anti-tumor response, break the immune surveillance, and promote tumor immune invasion [35]. It has been demonstrated that HHLA2 protein could be widely found in many human cancer tissues, such as breast, lung, thyroid, melanoma, pancreas, ovary, liver, bladder, colon, prostate, kidney, and esophagus cancers [23, 36]. Further studies demonstrated that higher expression level of HHLA2 in human lung cancer tissues significantly associated with EGFR mutation, higher intensities of TILs and PD-L1 status, suggesting an effective immunotherapy strategy for PD-L1-negative patients [37, 38]. Higher HHLA2 expression could also be found in triple negative breast cancer patients at stage I to III, and significantly associated with lymph node metastasis and poorer prognosis [23, 35]. Koirala et al. showed that HHLA2 was highly expressed in the majority of osteosarcoma tumors, and its expression level could be used to predict metastatic status and poorer survival of the patients [39].

In our present study, we reported that the higher HHLA2 expression could be found in human ccRCC tissues, both at the mRNA level from the TCGA data and at the protein level from the immunohistochemistry results, indicating that HHLA2 might be a novel immunosuppressive mechanism within the tumor microenvironment of human ccRCC. Our immunohistochemistry study and the clinical data analysis also showed that increased HHLA2 expression in human renal cancer tissues significantly associated with the poorer overall survival and advanced TNM stage of the patients, suggesting that abnormal expression of HHLA2 was involved in the oncogenesis and progression of human ccRCC. However, different from the results from other groups [24, 35], we could not find any significant relationship between HHLA2 expression level and the intensities of CD8^+^ T cell infiltration as well as the CD34^+^ intratumoral microvessels (data not shown). However, when we used the RNAi approach to establish the knockdown of HHLA2 expression in human ccRCC cell lines, we found that HHLA2 knockdown could significantly decreased the cell viability, migration ability, invasion ability, and induced the cell cycle arrest at G1 phase. The cellular studies further confirmed that the expressions of Cyclin D1, c-Myc and Cyclin E1 were significantly decreased after knockdown of HHLA2 expression.

In order to further investigate the regulatory mechanism of HHLA2 on cellular function and signal pathways in human ccRCC cell lines, we performed the Agilent lncRNA microarray analysis to explore the differentially expressed genes profiles between LV-HHLA2-sh1 and LV-NC groups. The co-down-regulated as well as the co-up-regulated gene profiles were analyzed, and among the top 20 pathways, we further investigated the epithelial-to-mesenchymal transition (EMT) upon knockdown of HHLA2 expression in ccRCC cell lines. Our results showed that the EMT marker E-cadherin expression was significantly increased, and N-cadherin as well as Vimentin was significantly decreased after knockdown of HHLA2 expression, suggesting that HHLA2 was involved in the cancer progression of human ccRCC by promoting EMT. It is noteworthy that many B7 family ligands were reported to have an important regulatory role in the EMT of cancer cells. For instance, PD-L1 has been confirmed to be an important mediator of EMT in certain human cancer tissues, such as lung cancer, colorectal cancer, esophageal cancer, and head and neck cancer [28, 40–42]. Moreover, other members such as B7-H3 and B7-H4, have also been suggested to promote EMT of cancer cells during cancer progression [43–45].

Collectively, our present study reported the clinical significance of abnormal HHLA2 expression in human ccRCC. Moreover, we also explored the contribution of HHLA2 to pathogenesis and progression of human ccRCC, and further confirmed that HHLA2 was involved in the promotion of EMT during cancer progression.

## Conclusions

Our present findings suggested that HHLA2 expression in human clear cell renal cell carcinoma is significantly associated with patient’s prognosis and promotes cancer progression, and could serve as important prognostic predictor and therapeutic target for this malignancy.

## Abbreviations

ccRCC: clear cell renal cell carcinoma
HHLA2: human endogenous retrovirus-H long terminal repeat-associating protein 2
TMIGD2: transmembrane and immunoglobulin domain containing 2
H&E: hematoxylin and eosin
FBS: fetal bovine serum
DMEM: Dulbecco’s modified Eagle’s medium
PBS: phosphate-buffered saline
shRNA: small hairpin RNA

## References

[1] Siegel RL, Miller KD, Jemal A (2018). Cancer statistics, 2018. CA Cancer J Clin.

[2] Rini BI, Campbell SC, Escudier B (2009). Renal cell carcinoma. Lancet.

[3] Lopez-Beltran A, Scarpelli M, Montironi R, Kirkali Z (2006). 2004 WHO classification of the renal tumors of the adults. Eur Urol.

[4] Rini BI, Rathmell WK, Godley P (2008). Renal cell carcinoma. Curr Opin Oncol.

[5] Mogami T, Harada J, Kishimoto K, Sumida S (2007). Percutaneous MR-guided cryoablation for malignancies, with a focus on renal cell carcinoma. Int J Clin Oncol.

[6] Rini BI (2011). Targeted therapy for patients with renal-cell carcinoma. Lancet Oncol.

[7] Cohen HT, McGovern FJ (2005). Renal-cell carcinoma. N Engl J Med.

[8] Quinn DI, Lara PN (2015). Renal-cell cancer-targeting an immune checkpoint or multiple kinases. N Engl J Med.

[9] Chen W, Hill H, Christie A, Kim MS, Holloman E, Pavia-Jimenez A, Homayoun F, Ma Y, Patel N, Yell P (2016). Targeting renal cell carcinoma with a HIF-2 antagonist. Nature.

[10] Nazzani S, Bazinet A, Karakiewicz PI (2018). Role of immunotherapy in kidney cancer. Curr Opin Support Palliat Care.

[11] Escudier B, Porta C, Eisen T, Belsey J, Gibson D, Morgan J, Motzer R (2018). The role of tivozanib in advanced renal cell carcinoma therapy. Expert Rev Anticancer Ther.

[12] Topalian SL, Hodi FS, Brahmer JR, Gettinger SN, Smith DC, McDermott DF, Powderly JD, Carvajal RD, Sosman JA, Atkins MB (2012). Safety, activity, and immune correlates of anti-PD-1 antibody in cancer. N Engl J Med.

[13] Liu M, Wu H, Shangguan D, Jiang Y, Li X, Liu S, Zhou B, Yin T, Gong Z (2018). Immunomodulatory therapies for renal cell carcinoma. Protein Pept Lett.

[14] McDermott DF, Huseni MA, Atkins MB, Motzer RJ, Rini BI, Escudier B, Fong L, Joseph RW, Pal SK, Reeves JA (2018). Clinical activity and molecular correlates of response to atezolizumab alone or in combination with bevacizumab versus sunitinib in renal cell carcinoma. Nat Med.

[15] Motzer RJ, Tannir NM, McDermott DF, Aren Frontera O, Melichar B, Choueiri TK, Plimack ER, Barthelemy P, Porta C, George S (2018). Nivolumab plus ipilimumab versus sunitinib in advanced renal-cell carcinoma. N Engl J Med.

[16] Santoni M, Massari F, Di Nunno V, Conti A, Cimadamore A, Scarpelli M, Montironi R, Cheng L, Battelli N, Lopez-Beltran A (2018). Immunotherapy in renal cell carcinoma: latest evidence and clinical implications. Drugs Context.

[17] Crispen PL, Sheinin Y, Roth TJ, Lohse CM, Kuntz SM, Frigola X, Thompson RH, Boorjian SA, Dong H, Leibovich BC (2008). Tumor cell and tumor vasculature expression of B7-H3 predict survival in clear cell renal cell carcinoma. Clin Cancer Res.

[18] Krambeck AE, Thompson RH, Dong H, Lohse CM, Park ES, Kuntz SM, Leibovich BC, Blute ML, Cheville JC, Kwon ED (2006). B7-H4 expression in renal cell carcinoma and tumor vasculature: associations with cancer progression and survival. Proc Natl Acad Sci USA.

[19] Fiegler N, Textor S, Arnold A, Rolle A, Oehme I, Breuhahn K, Moldenhauer G, Witzens-Harig M, Cerwenka A (2013). Downregulation of the activating NKp30 ligand B7-H6 by HDAC inhibitors impairs tumor cell recognition by NK cells. Blood.

[20] Wu F, Wang J, Ke X (2016). Knockdown of B7-H6 inhibits tumor progression and enhances chemosensitivity in B-cell non-Hodgkin lymphoma. Int J Oncol.

[21] Zhou Y, Xu Y, Chen L, Xu B, Wu C, Jiang J (2015). B7-H6 expression correlates with cancer progression and patient’s survival in human ovarian cancer. Int J Clin Exp Pathol.

[22] Zhao R, Chinai JM, Buhl S, Scandiuzzi L, Ray A, Jeon H, Ohaegbulam KC, Ghosh K, Zhao A, Scharff MD (2013). HHLA2 is a member of the B7 family and inhibits human CD4 and CD8 T-cell function. Proc Natl Acad Sci USA.

[23] Janakiram M, Chinai JM, Fineberg S, Fiser A, Montagna C, Medavarapu R, Castano E, Jeon H, Ohaegbulam KC, Zhao R (2015). Expression, clinical significance, and receptor identification of the newest B7 family member HHLA2 protein. Clin Cancer Res.

[24] Zhu Z, Dong W (2018). Overexpression of HHLA2, a member of the B7 family, is associated with worse survival in human colorectal carcinoma. Onco Targets Ther.

[25] Janakiram M, Shah UA, Liu W, Zhao A, Schoenberg MP, Zang X (2017). The third group of the B7-CD28 immune checkpoint family: HHLA2, TMIGD2, B7x, and B7-H3. Immunol Rev.

[26] Chen L, Sun J, Wu H, Zhou S, Tan Y, Tan M, Shan B, Lu B, Zhang X (2011). B7-H4 expression associates with cancer progression and predicts patient’s survival in human esophageal squamous cell carcinoma. Cancer Immunol Immunother.

[27] Chen LJ, Zheng X, Shen YP, Zhu YB, Li Q, Chen J, Xia R, Zhou SM, Wu CP, Zhang XG (2013). Higher numbers of T-bet(+) intratumoral lymphoid cells correlate with better survival in gastric cancer. Cancer Immunol Immunother.

[28] Chen L, Xiong Y, Li J, Zheng X, Zhou Q, Turner A, Wu C, Lu B, Jiang J (2017). PD-L1 expression promotes epithelial to mesenchymal transition in human esophageal cancer. Cell Physiol Biochem.

[29] Chen L, Zhai W, Zheng X, Xie Q, Zhou Q, Tao M, Zhu Y, Wu C, Jiang J (2018). Decreased IFIT2 expression promotes gastric cancer progression and predicts poor prognosis of the patients. Cell Physiol Biochem.

[30] Chen L, Feng J, Wu S, Xu B, Zhou Y, Wu C, Jiang J (2018). Decreased RIG-I expression is associated with poor prognosis and promotes cell invasion in human gastric cancer. Cancer Cell Int.

[31] Chen L, Chen J, Xu B, Wang Q, Zhou W, Zhang G, Sun J, Shi L, Pei H, Wu C (2015). B7-H3 expression associates with tumor invasion and patient’s poor survival in human esophageal cancer. Am J Transl Res.

[32] Guo H, Liu J, Ben Q, Qu Y, Li M, Wang Y, Chen W, Zhang J (2016). The aspirin-induced long non-coding RNA OLA1P2 blocks phosphorylated STAT3 homodimer formation. Genome Biol.

[33] Mager DL, Hunter DG, Schertzer M, Freeman JD (1999). Endogenous retroviruses provide the primary polyadenylation signal for two new human genes (HHLA2 and HHLA3). Genomics.

[34] Zhu Y, Yao S, Iliopoulou BP, Han X, Augustine MM, Xu H, Phennicie RT, Flies SJ, Broadwater M, Ruff W (2013). B7-H5 costimulates human T cells via CD28H. Nat Commun.

[35] Janakiram M, Chinai JM, Zhao A, Sparano JA, Zang X (2015). HHLA2 and TMIGD2: new immunotherapeutic targets of the B7 and CD28 families. Oncoimmunology.

[36] Chen D, Chen W, Xu Y, Zhu M, Xiao Y, Shen Y, Zhu S, Cao C, Xu X (2019). Upregulated immune checkpoint HHLA2 in clear cell renal cell carcinoma: a novel prognostic biomarker and potential therapeutic target. J Med Genet.

[37] Cheng H, Borczuk A, Janakiram M, Ren X, Lin J, Assal A, Halmos B, Perez-Soler R, Zang X (2018). Wide expression and significance of alternative immune checkpoint molecules, B7x and HHLA2, in PD-L1-negative human lung cancers. Clin Cancer Res.

[38] Cheng H, Janakiram M, Borczuk A, Lin J, Qiu W, Liu H, Chinai JM, Halmos B, Perez-Soler R, Zang X (2017). HHLA2, a new immune checkpoint member of the B7 family, is widely expressed in human lung cancer and associated with EGFR mutational status. Clin Cancer Res.

[39] Koirala P, Roth ME, Gill J, Chinai JM, Ewart MR, Piperdi S, Geller DS, Hoang BH, Fatakhova YV, Ghorpade M (2016). HHLA2, a member of the B7 family, is expressed in human osteosarcoma and is associated with metastases and worse survival. Sci Rep.

[40] Kim S, Koh J, Kim MY, Kwon D, Go H, Kim YA, Jeon YK, Chung DH (2016). PD-L1 expression is associated with epithelial-to-mesenchymal transition in adenocarcinoma of the lung. Hum Pathol.

[41] Ock CY, Kim S, Keam B, Kim M, Kim TM, Kim JH, Jeon YK, Lee JS, Kwon SK, Hah JH (2016). PD-L1 expression is associated with epithelial–mesenchymal transition in head and neck squamous cell carcinoma. Oncotarget.

[42] Zhi Y, Mou Z, Chen J, He Y, Dong H, Fu X, Wu Y (2015). B7H1 expression and epithelial-to-mesenchymal transition phenotypes on colorectal cancer stem-like cells. PLoS ONE.

[43] Lou Y, Diao L, Cuentas ER, Denning WL, Chen L, Fan YH, Byers LA, Wang J, Papadimitrakopoulou VA, Behrens C (2016). Epithelial–mesenchymal transition is associated with a distinct tumor microenvironment including elevation of inflammatory signals and multiple immune checkpoints in lung adenocarcinoma. Clin Cancer Res.

[44] Kang FB, Wang L, Jia HC, Li D, Li HJ, Zhang YG, Sun DX (2015). B7-H3 promotes aggression and invasion of hepatocellular carcinoma by targeting epithelial-to-mesenchymal transition via JAK2/STAT3/Slug signaling pathway. Cancer Cell Int.

[45] Wu H, Wang X, Mo N, Zhang L, Yuan X, Lu Z (2018). B7-homolog 4 promotes epithelial-mesenchymal transition and invasion of bladder cancer cells via activation of nuclear factor-kappaB. Oncol Res.

